# Degradation Mechanisms for GaN and GaAs High Speed Transistors

**DOI:** 10.3390/ma5122498

**Published:** 2012-11-27

**Authors:** David J. Cheney, Erica A. Douglas, Lu Liu, Chien-Fong Lo, Brent P. Gila, Fan Ren, Stephen J. Pearton

**Affiliations:** 1 Department of Electrical and Computer Engineering, University of Florida, Gainesville, FL 32611, USA; E-Mail: djcheney@ufl.edu; 2 Department of Materials Science and Engineering, University of Florida, Gainesville, FL 32611, USA; E-Mails: ericaadouglas@gmail.com (E.A.D.); bgila@mse.ufl.edu (B.P.G.); 3 Department of Chemical Engineering, University of Florida, Gainesville, FL 32611, USA; E-Mails: luliu@ufl.edu (L.L.); cflo@ufl.edu (C.-F.L.); ren@che.ufl.edu (F.R.)

**Keywords:** degradation, stress, aging, HBT, HEMT

## Abstract

We present a review of reliability issues in AlGaN/GaN and AlGaAs/GaAs high electron mobility transistors (HEMTs) as well as Heterojunction Bipolar Transistors (HBTs) in the AlGaAs/GaAs materials systems. Because of the complex nature and multi-faceted operation modes of these devices, reliability studies must go beyond the typical Arrhenius accelerated life tests. We review the electric field driven degradation in devices with different gate metallization, device dimensions, electric field mitigation techniques (such as source field plate), and the effect of device fabrication processes for both DC and RF stress conditions. We summarize the degradation mechanisms that limit the lifetime of these devices. A variety of contact and surface degradation mechanisms have been reported, but differ in the two device technologies: For HEMTs, the layers are thin and relatively lightly doped compared to HBT structures and there is a metal Schottky gate that is directly on the semiconductor. By contrast, the HBT relies on pn junctions for current modulation and has only Ohmic contacts. This leads to different degradation mechanisms for the two types of devices.

## 1. Introduction

The basic unit of failure in Si device technology is the FIT (Failure unit), defined as 1 failure/10^9^ device hours. For 100 devices on test, a failure rate of 1000 FIT would mean there would be only 1 failure in 1 year. Given that a relatively small number of devices will actually show failure, it is critical to both enhance the failure rate through accelerated testing (the five common stresses used are temperature, voltage, current, humidity and temperature cycling) and to treat the resulting reliability data correctly. Under accelerated aging conditions, different failure mechanisms may be accelerated by different amounts for the same applied stress.

The global reliability data for compound devices shows no obvious improvement in 20 years [1]. In the past 40 years, the focus of GaAs-based device technologies shifted from Metal Semiconductor Field Effect Transistors (MESFETs) to various types of High Electron Mobility Transistors (HEMTs) and then to Heterojunction Bipolar Transistors (HBTs). More recently, GaN-based HEMTs have received much attention for use in high power and high frequency applications due to large energy band-gaps, great electron mobility, high breakdown voltages, and considerable 2-D electron gas densities as compared to their GaAs counterparts. Employment of GaN HEMTs for high power radar systems will require devices to be subjected to large-signal RF while being driven into saturation, resulting in devices experiencing high electric fields and high current densities. Impressive mean-time-to-failure values of greater than 10^7^ h have been reported at operating temperatures below 200 °C, with activation energies ranging from 0.18 eV to 2 eV [1,2,3,4,5,6,7,8,9]. However, these studies neglect the effect of high electric field and current on device lifetime. Additionally as the demand increases for faster data communication at higher frequencies, scaling of the gate will be driven below 0.2 µm.

Much of the reliability work to date on compound semiconductors was driven by a heavy emphasis on defense funding for the necessary research, with few large commercial applications until the cell phone market exploded. By contrast, in the Si industry, the basic device has always been the Metal Oxide Semiconductor Field Effect Transistor (MOSFET). In the initial work on GaAs MESFETs, the main reliability issues were due to diffusion of gate metals with the underlying semiconductor, typically referred to as gate sinking and also the need for effective surface passivation to prevent creation of surface states that lead to unwanted depletion effects and carrier trapping. With the shift in emphasis to epitaxial layered structures such as HEMTs and HBTs in the 1990’s, issues such as dopant diffusion (especially the base dopant in HBTs), hydrogen passivation and contamination (in both HEMTs and HBTs) became important. As the requirements for high power operation and integration level increased, field-driven failure mechanism related to trap creation, surface breakdown and contact failure were all identified as being important and in addition, the failure rate of passive components began to be an issue. With the introduction of GaN-based transistors as well as the continued maturation of the GaAs technologies [10,11,12,13,14,15,16], defect reduction and a focus on improved capacitors and interconnects were areas of focus. The continual introduction of new materials in compound semiconductor technologies was a drawback compared to Si, where problems such as mobile ion migration, electromigration, hot carrier effects, time dependent dielectric breakdown, corrosion, electrostatic discharge and soft errors could be systematically solved and protocols and models developed for their understanding and mitigation. This allowed the Si industry to focus on designing and building-in reliability at the wafer level and to introduce defect reduction efforts and strict process controls. The recent introduction of new materials in Si technologies, such as Cu for interconnects and high and low K materials for gates and interconnects probably means some new areas of reliability concern, but the huge experience base should minimize unexpected problems.

In Si MOS devices, time dependent failure mechanisms include surface charge accumulation or injection, dielectric breakdown, electro-migration, contact degradation and corrosion due to contamination. In compound semiconductors, there are added issues of local regions of non-stoichiometry that affect field distributions and increase recombination, oxidation of AlGaAs or AlGaN, high densities of dislocations and other extended defects in some structures and high surface state densities. In GaAs devices, it is important to pay close attention to voltage and current acceleration stress mechanisms. Many studies in Si indicate the reaction rate (*R*) of the failure mechanism is proportional to a power of the applied voltage (*V*) as well as temperature, *i.e.*,

(1) $$R\left(T,V\right)=R_{O}\left(T\right)V^{\gamma \left(T\right)}$$

where the coefficient *R*_O_(*T*) is an Arrhenius function of temperature (*T*) and the power dependence varies between 1 and 4.5. This determines how much acceleration upon increasing the bias voltage used during stressing. If dielectric breakdown is the dominant failure mode, then at a given field, a fraction of the devices will fail in a short time, with no additional failures until an increased field is applied.

The failure rate at operating temperatures is extrapolated from the failure rate under thermally accelerated stress conditions through the Arrhenius equation. This equation is limited to acceleration of failure mechanisms due to physio-chemical reactions [1,2,10]. With the bulk of reliability predictions focusing on Arrhenius extrapolations of device lifetime, they do not capture failure mechanisms that govern degradation under DC or RF operation that cannot be accelerated by elevated temperatures [1].

## 2. Typical Accelerated Stress Protocols

GaAs and GaN reliability studies have typically focused on temperature enhancement of failure to get adequate statistics (the so-called 3 temperature test is common). In this case, devices are tested at different temperatures (*T*) and an activation energy (*Ea*) extracted from the relationship

(2) $$\ln(t_{1}/t_{2})=Ea/k(1/T_{1}-1/T_{2})$$

where *t*_1,2_ are time to failure at temps *T*_1,2_ and k is Boltzmann’s constant. However, it is necessary to use voltage and current-enhancement at realistic operating temperatures to avoid the shortcomings of the Arrhenius extrapolation and hence use relations of the type shown in Equation (1). When one simply uses temperature as the accelerant, serious errors in estimating device lifetime are possible. If more than one degradation mechanism is present, testing at high temperature and then extrapolating back to the normal device operating temperature may miss the real mechanism that limits the device lifetime at that particular temperature. This seems to be generally understood and there is increasing use of voltage, current and RF stressing at temperatures closer to the actual device operating temperature. Actual field returns may be dominated by other factors, including ESD, capacitor defects, assembly/packaging issues, rather than device degradation.

**Figure 1:**
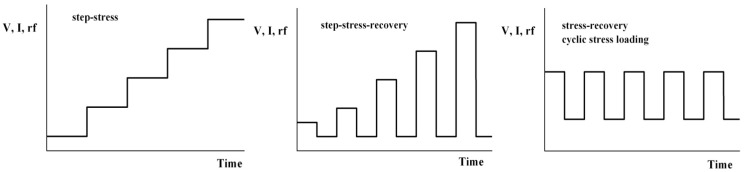
Examples of typical time-dependence of stressing protocols for III-V electronic devices. The parameter that is varied with time can be voltage, current, RF power, amongst others.

**Figure 2:**
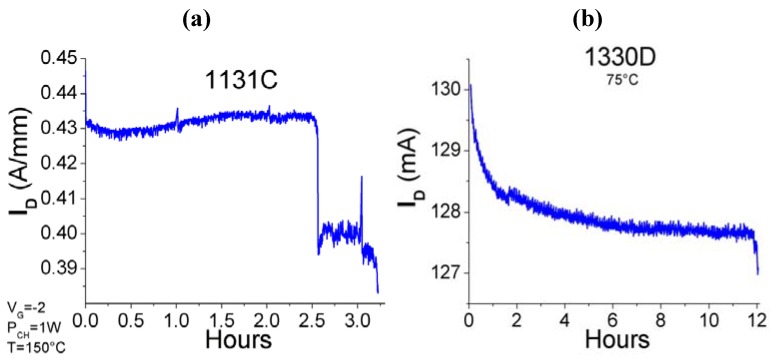
Examples of catastrophic (**a**) and gradual degradation (**b**) of GaN high electron mobility transistors (HEMTs) during drain voltage step stressing at 150 °C (**a**) or 75 °C (**b**).

Some typical approaches to stressing at fixed temperature are illustrated in Figure 1, which shows the so-called step stress approach, in which the parameter of interest such as voltage, current or RF power, is stepped in some fashion, either monotonically or with a recovery period in between. The latter types of stressing are useful for detecting the presence of traps that empty during the recovery period. Figure 2 shows examples of devices degrading rapidly or gradually during such step-stressing of the drain voltage.

In compound semiconductor device technology, it is being increasingly realized that one must look for not only the dominant factor causing degradation but also whether there is recovery during reverse biasing (trap generation), the role of the ambient (in case of oxidation or hydrogen effects), the electric field distributions (including inspection of non-uniformity in gate dimensions). A complete description of the device degradation might also include visual inspection, DC, RF, base noise spectra (f, V), check for high leakage. It is common to define failure as a change of only 10%–20% in some key performance parameter, such as, for example, a drop of this magnitude in HBT gain.

## 3. GaN Degradation Mechanisms

As the device design and material processing technology for AlGaN/GaN HEMTs has matured over the years, several failure mechanisms that limited device lifetime have been addressed and improved. These mechanisms can be grouped together into three main categories that affect lifetime: Contact degradation, hot electron effect, and inverse piezoelectric effect. Both Schottky and Ohmic contacts have shown excellent stability below 300 °C [11]. Piazza *et al.* have reported an increase in contact resistance and passivation cracking due to Ga out-diffusion and Au inter-diffusion after a 100 h thermal storage test stress at 340 °C [17]. Nickel based Schottky contacts have been shown to form nickel nitrides on GaN at annealing temperatures as low as 200 °C, resulting in a significant decrease in Schottky barrier height [18,19].

The observed current collapse and gate lag in AlGaN/GaN HEMTs under high voltage and high current operation have been attributed to hot electrons. These are electrons that have been accelerated in a large electric field, resulting in very high kinetic energy, which can result in trap formation. Creation of traps can occur in both the AlGaN layer and the buffer, leading to reversible degradation of transconductance and saturated drain current [2,5,11]. GaN is a piezoelectric material and under high bias conditions, the electric field induces additional tensile stress to the already strained AlGaN layer [20,21,22,23,24,25,26]. Several authors have shown that upon reaching a “critical voltage”, irreversible damage to the device occurs resulting in defect formation through which electron leakage can occur [10,11,12,13,14].

### 3.1. Hot-Carriers and Trap Generation

Permanent device degradation after high V_DG_ stress under on-state conditions has been attributed to the presence of hot electrons. In GaAs-based devices, hot electrons generate holes which are accumulated by the gate and result in a negative shift in *V*_T_ [25,26,27]. Typically, I_G_ is used to derive the field-acceleration laws for failure. Impact ionization, however, is negligible in GaN HEMTs. This is due to the fact that tunneling injection dominates gate current, preventing gate current from being used as an indicator for hot electron degradation [1,4]. However, these hot electrons likely lead to trap generation at the AlGaN/GaN interface and/or at the passivation GaN cap interface. As in GaAs and InP based HEMTs, traps lead to an increase in the depletion region between the gate and the drain, ultimately resulting in an increase in drain resistance and subsequently a decrease in saturated drain-source current. Comparatively, under reverse bias or so-called off-state conditions the degradation is greatly reduced due to the reduction of electrons present in the channel. Sozza *et al.* showed that GaN/AlGaN/GaN HEMTs that underwent a 3000 h on-state stress resulted in an increase in surface traps with an activation energy of about 0.55 eV [3,8]. On the other hand, devices stressed under off-state conditions saw a very small increase in traps.

Meneghesso *et al.* have employed the use of electroluminescence (EL) to study the effect of hot-carriers and its dependence on stress conditions [2]. Uniform EL emission was observed along the channel for devices stressed at *V*_GS_ = 0 V and *V*_DS_ = 20 V, which is due to hot electrons. However, there is no presence of hot spots or current crowding. On the other hand, under OFF state conditions with *V*_GS_ = −6 V and *V*_DS_ = 20 V (resulting in a *V*_GD_ = −26 V), the EL emission from the channel is not uniform. These hot spots may be due to injection of electrons from the gate into the channel. Due to the high bias conditions, the electrons acquire enough energy to give rise to photon emission.

### 3.2. Contact Degradation

Contact degradation and gate sinking are significant degradation mechanisms at elevated temperatures in GaAs and InP based HEMTs. This has not yet proven to be a significant issue with AlGaN/GaN HEMTs at temperatures below 400 °C for Pt/Au Schottky contacts and Ti/Al/Pt/Au annealed Ohmic contacts [1,11,28,29,30,31,32]. An increase in Schottky barrier height was observed for Ni/Au Schottky contacts after dc stress at elevated junction temperatures (200 °C) [1,11,33,34,35,36]. This was due to a consumption of an interfacial layer between the Schottky contact and the AlGaN layer. Though the resulting positive shift in the Schottky barrier height, and thus the pinch-off voltage, is ideal, the subsequent change in I_DSS_ is not favorable. Unstressed devices were subjected to an anneal after the Schottky contact was deposited in order to decrease the interfacial layer between the gate and semiconductor. Devices that underwent the gate anneal showed 50% less degradation during a 24 h stress test as opposed to devices that did not receive a gate anneal [1,11]. Thermal storage tests up to 2000 h on Ti/Al/Ni/Au ohmic contacts at and above 290 °C showed an increase in contact resistance as well as surface roughness due to growth of Au-rich grains that ultimately led to cracks in passivation [33,34,35,36,37,38,39,40]. The two primary degradation mechanisms were determined to be Au inter-diffusion within the metal layers and Ga out-diffusion from the semiconductor into the metallic compounds. Similar degradation was observed after dc stress tests that resulted in junction temperatures equivalent to the thermal storage tests. Due to the high power capability of AlGaN/GaN HEMTs, proper temperature management is crucial in order to optimize device performance under high current and high voltage operation [41,42,43,44,45,46,47,48]. Self heating of devices can ultimately result in poor device performance through contact degradation. Reliability of contacts is highly dependent upon both metal schemes as well as processing during fabrication.

### 3.3. Inverse Piezoelectric Effect

Several research groups have shown that high reverse bias on the gate results in the generation of defects that provide a path for gate current leakage [1,11,33]. This defect formation mechanism is a result of the inverse piezoelectric effect. Due to the fact that GaN and AlGaN are intrinsically piezoelectric materials, the presence of high electric fields will result in an increase in stress within the GaN and AlGaN layers. AlGaN is lattice mismatched to GaN, resulting in significant tensile strain, even in the absence of an electric field. If under electric stress the elastic energy within the AlGaN/GaN layers surpasses a critical value, the strained layer will relax through crystallographic defect formation. It is possible that the defects could be electrically active and result in device degradation [33].

J. Joh *et al.* have established that *I*_D_ and *I*_G_ degradation under high reverse gate bias occurs at a critical voltage, typically above 20 V on *V*_DG_ [38]. This is also correlated with a sharp rise in both source and drain resistance as well as a positive shift in *V*_T_. However, the critical voltage for devices can deviate substantially within one wafer, though adjoining devices appear to exhibit similar performance. The critical voltage corresponds to a threshold field that leads to immediate device degradation if it is exceeded. The degradation exhibits a time dependence at lower fields, being slower the further below critical voltage that the device is biased. The broad distribution of critical voltage observed, ranging from *V*_DG_ of ~15 V to ~30 V, has been attributed to slow changes within the substrate or epi-layer growth over the wafer [36,37,38,39,40,41]. As mentioned above, hot electrons are generated exponentially as the field increases and only linearly with current [1,4]. Though stress experiments in the high power state have shown that increasing *I*_Dstress_ does not significantly accelerate degradation, it was found that the critical voltage for reverse bias stress in which *I*_Goff_ dramatically increases is dependent upon the *I*_Dstress_, as *V*_crit_ increases with increasing *I*_Dstress_ [33,38]. It is evident from this result that hot electrons are not the driving degradation mechanism for this stress condition. To verify the inverse piezoelectric effect, TEM cross sections were studied by Chowdhury et al after stressing with *V*_DS_ = 40 V and *I*_D0_ = 250 mA/mm at various base-plate temperatures, which corresponded to junction temperatures of 250 °C, 285 °C, and 320 °C based on device modeling [10]. Unstressed devices showed no evidence of pits or cracks near the edge of the Schottky contact. However, all stressed devices showed evidence of pit-like defects on the drain side of the gate. The depth of the pit was about 10nm, and remained within the AlGaN layer. Crack-like defects were observed in a few of the stressed devices, and appeared to originate at the bottom of the pit defect, extending to the heterointerface of the AlGaN/GaN layer and occasionally into the GaN buffer. As the junction temperature increased, the time after which the crack appeared decreased, developing within 6 h at a temperature of 320 °C. Gate metal was also observed to diffuse ~2 nm into the defect crack. The formation of the crack was hypothesized by Chowdhury *et al.* to originate in the deepest points in the defect pit and spread along the gate width, thus explaining the presence of cracks in very shallow defect pits [10].

del Alamo *et al.* have postulated that the inverse piezoelectric effect is solely an electric field driven degradation mechanism due to the fact that it is the induced mechanical stress that results in the relaxation of the AlGaN layer [11,39]. It has also been hypothesized by the del Alamo group that current should not drive this mechanism, except for indirect self heating that would accelerate degradation of the device. Device design that affects the profile of the electric field on the drain side of the gate will also, in turn, impact the critical voltage [39].

Sarua *et al.* have investigated the effect of piezoelectric strain in AlGaN/GaN FETs under bias with micro-Raman spectroscopy [14]. Devices implemented 30 nm of Al_0.25_Ga_0.75_N on 1.2 µm of undoped, insulating GaN on insulating 4H-SiC with 2 µm × 50 µm (source-drain gap = 4.8 µm, gate length = 1.2 µm). It was confirmed with 2D finite element simulations that a pinched-off device at *V*_DS_ = 20 V results in a peak electric field on the drain side of the gate within the AlGaN layer. However, the *z* component of the electric field extended down into the GaN layer [14]. Fe doping of the GaN buffer layer is often used in order to improve the control of short-channel effects in HFETs [17]. It was later shown by Sarua *et al.* that Fe doped GaN, which raises the acceptor concentration and decreases the depletion width in the GaN buffer, confines the *z* component of the electric field to the AlGaN/GaN interface [14]. Self-heating will occur under high power stress conditions, which results in a compressive thermal strain/stress [42,43]. It is possible that due to the mitigation of the piezoelectric stress by the thermal stress, slower device degradation was seen in devices stressed under the high power state as opposed to off-state condition. This is in contrast to the hypothesis of del Alamo *et al*. that higher temperatures will result in an acceleration of device degradation [11,44,45].

Other issues can lead to additional compressive and tensile strains on the underlying epitaxial layers, including SiN passivation, which is used extensively to minimize surface traps on the AlGaN surface. Mastro *et al.* reported the simulated effects of non-uniform strain due to SiN passivation [18]. Typically, SiN has a relatively small magnitude of stress as compared to the tensile strain present in the AlGaN layer due to lattice mismatch. The strain in SiN is highly dependent on processing conditions, *i.e.*, thickness, frequency of the plasma during PECVD, pressure, and temperature. When deposited on the device, variations and discontinuities can increase the stress fields. For instance, the opening at the edge of the gate metal will result in a force on the AlGaN which will be perpendicular to the gate edge and parallel to the surface of the AlGaN [18]. It was predicted by Mastro *et al.* that as the gate length decreases, the magnitude of the strain fields increases. This effect on gate length is of great importance due to the desire to continuously scale down the dimensions of the devices.

**Figure 3:**
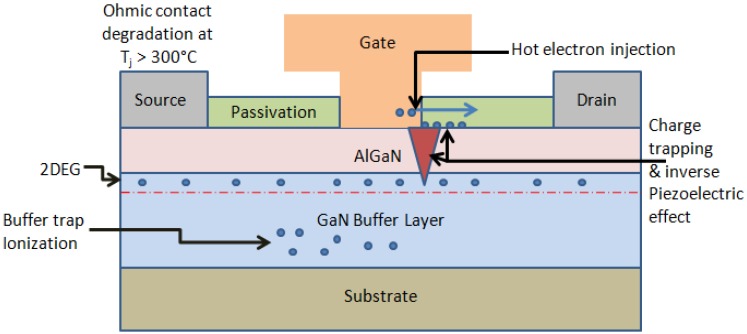
Schematic of degradation mechanisms in AlGaN/GaN HEMTs.

Figure 3 summarizes the reported degradation mechanisms in AlGaN/GaN HEMTs during electrical stressing at temperatures up to the typical operating temperature.

## 4. DC Stress Testing of AlGaN/GaN HEMTs

**Figure 4:**
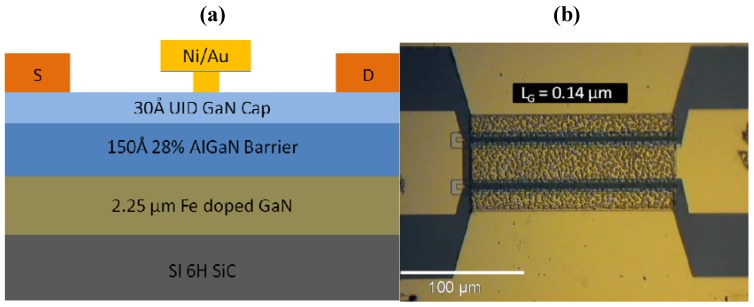
(**a**) Cross-section diagram of AlGaN/GaN HEMT; (**b**) Optical microscope image of device with gate length (*L*_G_) of 0.14 µm. Reprinted with permission from [46]. Copyright 2012 Elsevier.

**Figure 5:**
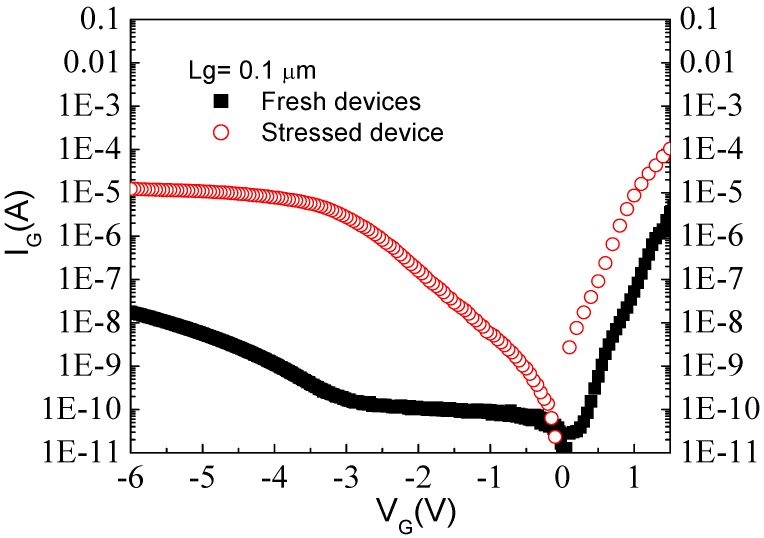
Gate current *versus* voltage before and after high reverse gate bias step stress for AlGaN/GaN HEMT.

To study the effect of high electric field on GaN HEMTs, off-state step-stresses were performed in the dark at room temperature with the gate biased up to −100 V reverse gate voltage at various fixed source-drain bias using an HP 4156C semiconductor parameter analyzer. Figure 4 shows the device cross section and an optical microscope image. As the gate voltage is stepped in 1 V increments for 1 min per step from −10 V to −42 V, gate leakage current in AlGaN/GaN HEMTs with Ni/Au-based gate metallization is seen to steadily increase until the critical voltage (*V*_CRI_) is reached, resulting in a permanent increase in gate leakage current several orders of magnitude (Figure 5). This sharp rise in current has been attributed to the inverse piezoelectric effect, in which the tensile strain in the AlGaN layer reaches a critical point whereby defect formation begins and subsequent lattice relaxation can occur. Even though a large increase in gate current is observed after stress, this is accompanied by a small decrease (~20%) in the drain current, due to an increase in on-state resistance. Similarly, the transfer characteristics typically show a reduction in maximum transconductance of ~40 mS/mm as a result of the gate bias stress cycle and a shift in threshold voltage of ~0.6 V.

**Figure 6:**
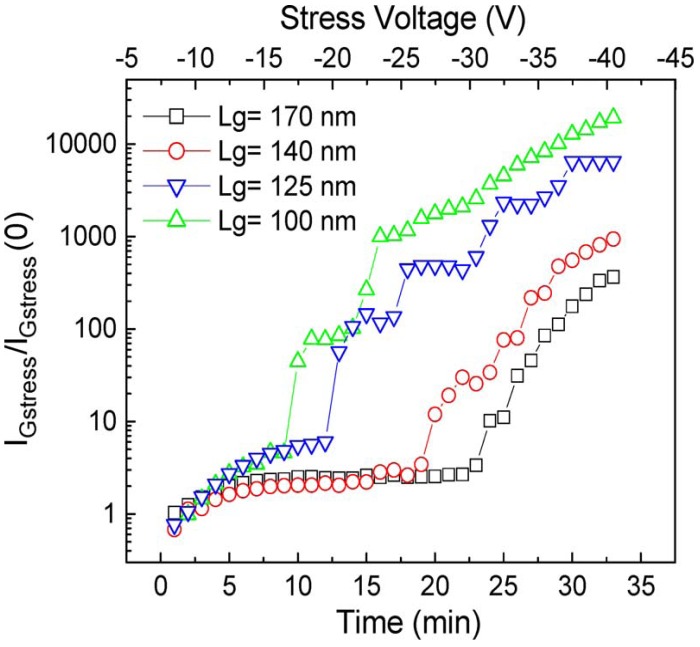
Gate current vs voltage before and after high reverse gate bias step stress for AlGaN/GaN HEMT.

Sub-micron gate lengths ranging from 0.1 to 0.17 µm were step stressed from −10 V to −42 V in the same manner. While a linear dependence of gate length on *V*_CRI_ is observed (Figure 6), ATLAS/Blaze simulations confirm that degradation occurs at a critical maximum electric field in the channel, ~1.8 MV/cm. This is consistent with previously published results indicating that failure is due to electric field induced tensile strain. Photoluminescence (PL) was performed on the degraded devices. The PL spectrum of an ungated, open area of an unstressed HEMT was measured. GaN showed a peak centered at 366 nm while the AlGaN peak was centered at 352 nm. After stressing the device, PL spectra showed a decrease in intensity in both peaks, but no additional defect peaks appeared. This degradation is indicative of the formation of non-radiative centers in both the GaN and AlGaN layers [47,48,49,50,51,52].

## 5. Effect of Field Plates on Stressing of AlGaN/GaN HEMTs

**Figure 7:**
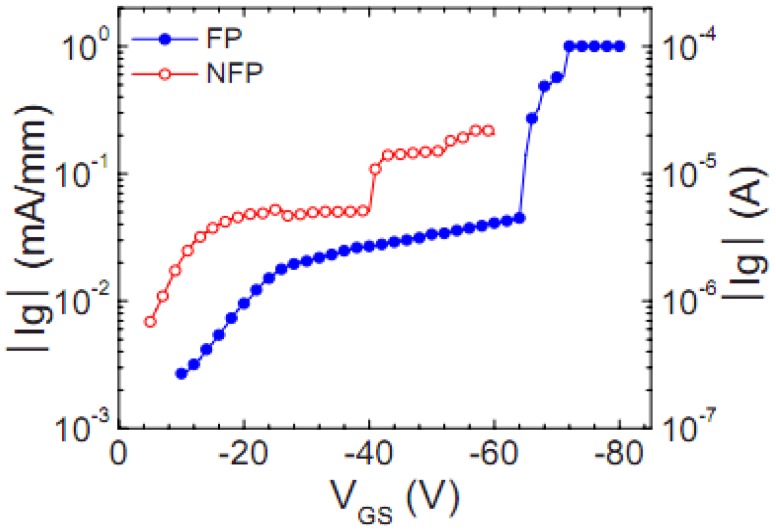
Off-state gate currents as a function of *V*_GS_ for AlGaN/GaN HEMT with (FP) and without source field plate (NFP).

To improve the off-state breakdown voltage, a source field plate over the gate electrode was fabricated to reduce the peak electric field at the drain side of the gate edge. This resulted in a significant increase in *V*_CRI_ from −40 V to −65 V (Figure 7). Transmission electron microscopy (TEM) images indicated the presence of Ni from the gate metal stack interacting with the underlying nitride layer in close proximity to a threading dislocation

Figure 7 shows the typical step-stress results of more than 30 HEMTs with and without the source field plate; the gate current, *I*_G_, has been plotted as a function of the stressed gate voltage. The devices were step stressed in −1 V increments for 1 min at each increment, while grounding the source electrode and maintaining +5 V to the drain. The typical critical voltages of HEMTs without the source field plate were around −40 V [53]. This value increased to around −65 V for the HEMTs with the source field plate. This increase of the critical voltage for the HEMT with the source field plate was attributed to the reduction in electric field on the drain side of the gate edge, consistent with the simulated electric-field results [54].

## 6. Role of Gate Metal in Failure of AlGaN/GaN HEMTs.

**Figure 8:**
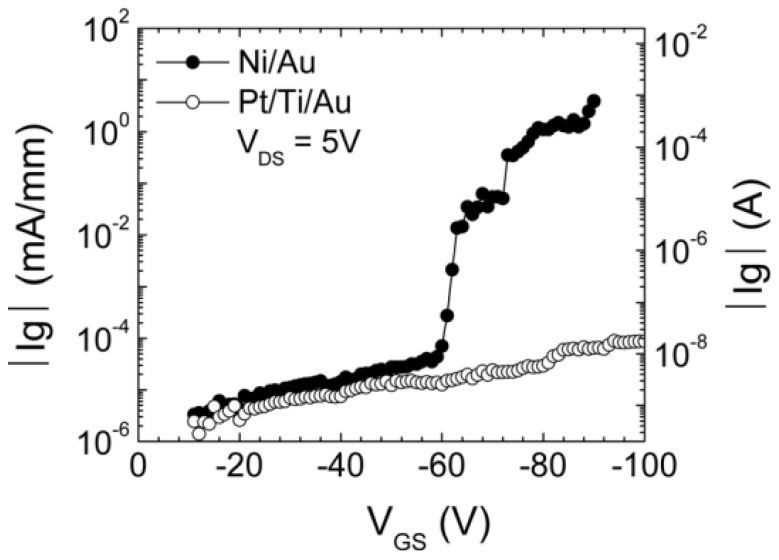
Off-state gate currents as a function of gate metal for AlGaN/GaN HEMTs fabricated with Ni/Au or Pt/Ti/Au gate metallization.

Fifteen separate HEMTs with Ni/Au or Pt/Ti/Au gate metallization wafers were stressed for 60 s at each gate voltage step, while grounding the source electrode and maintaining +5 V on the drain. The stress started at −10 V of gate voltage and the voltage step was kept at 1 V increments. As shown in Figure 8, the HEMTs with Ni/Au gate metallization exhibited a critical voltage around −55 V. However, there was no critical voltage observed for the HEMT with the Pt/Ti/Au gate metallization up to −100 V, which was limited by the instrument used in this experiment. This suggested that the use of Pt based gate metallization could extend the operating bias conditions and improve the device reliability. The Schottky barrier height and ideality of the Pt/Ti/Au were 1.23 eV and 1.21 eV, respectively, which did not exhibit noticeable changes as a result of the bias stressing. On the contrary, the HEMTs with Ni/Au gate metallization showed significantly higher gate reverse bias leakage current and much lower breakdown voltage. The forward gate characteristics of the Ni/Au gate contact appeared very leaky after the stress and the Schottky height reduced from 1.09 to 0.66 V after stress. While Pt displays superior stability, there is an issue of stress in the metal that often leads to peel-off of the contact metal [55,56,57,58,59,60].

## 7. RF Stress Tests of AlGaN/GaN HEMTs.

**Figure 9:**
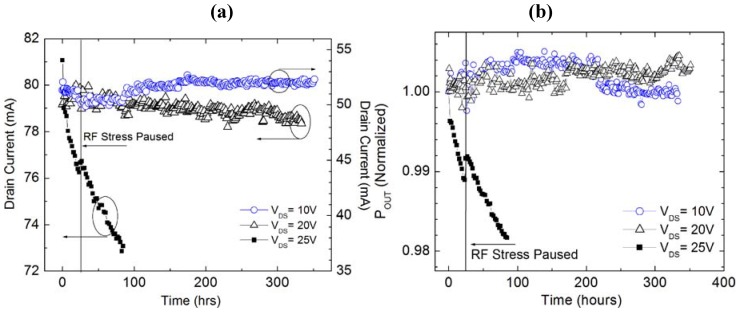
(**a**) Drain current and (**b**) output power during RF stress at 10 GHz under 3 dB compression of 0.125 µm gate length AlGaN/GaN HEMT. Reprinted with permission from [61]. Copyright 2011 Electrochemical Society.

Devices with a 0.125 µm gate length were biased under Class AB conditions with a quiescent drain current of 200 mA/mm at drain bias of 10 V, 20 V, and 25 V. RF stress up to 350 h was performed at 10 GHz, driven into 3 dB compression and a baseplate temperature of 30 °C. As can be seen in Figure 9, both POUT and IDS showed little to no degradation for drain bias of 10 V and 20 V.

However, rapid permanent degradation is present at a drain bias of 25 V. A small recovery in both *P*_OUT_ and *I*_DS_ is present after pausing at ~25 h to obtain mid-stress device characteristics. Operating at a drain bias of 25 V is above the threshold necessary for the onset of degradation and is consistent with the observed critical voltage during dc stress. Small degradation in saturated drain current (*I*_DSS_), less than 10%, occurred for all three drain bias conditions after stress and is consistent with prior reports on RF reliability [54]. For the lowest drain bias of 10 V, a small increase (~3%) in PAE and *I*_DS_ (~1 mA) was observed during RF stressing for 350 h, and may indicate that device burn-in is necessary in order to stabilize and improve RF device performance at lower bias conditions. Similarly, minimal degradation in PAE, *P*_OUT_, gain, and transconductance occurred for a drain bias of 20 V. An increase in threshold voltage (*V*_TH_) was observed for all stress conditions, with the largest Δ*V*_TH_ of 0.37 V at a drain bias of 25 V, indicating significant degradation of the Schottky contact. High dc bias stress of these devices, as well as others, have shown a consumption of the oxide layer present between the Schottky contact and underlying semiconductor layer, and has been correlated to an increase in threshold voltage and decrease in *I*_DSS_ [55].

Additionally, the Schottky barrier height reduced from 673 mV to 602 mV after stress at *V*_DS_ = 25V. Even though device characteristics for drain bias at or below 20 V do not show significant RF degradation, the gate current-voltage sweeps with *V*_DS_ = 0 V indicate that the Schottky contact suffered degradation, with almost two orders of magnitude increase in gate leakage current. Even though the stress at a drain bias of 25 V was performed for a shorter duration, only ~85 h, gate leakage current shows the largest increase with almost three orders of magnitude rise in current.

**Figure 10:**
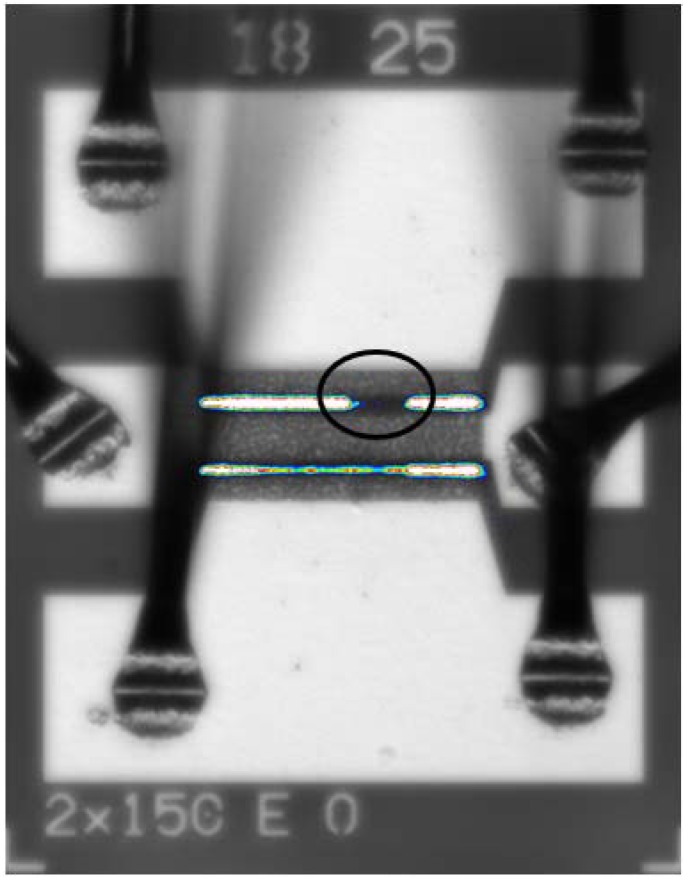
Electroluminescence of AlGaN/GaN HEMT after stress at *V*_DS_ = 25 V and Class AB operation. Circled region indicates area of channel that has an increase in non-radiative trap formation after stress. Uniform EL emission during forward bias was observed prior to stress. Photoluminescence spectra were taken at the circled region.

Prior to stress, devices exhibited uniform EL emission under forward bias. However, points of gate leakage appeared with gate bias of −10 V. After RF stress, gate leakage paths were not present on the same channel as present prior to stress (Figure 10) [55]. It appears from EL emission that degradation due to RF stress may be localized along the channel on the right. Additionally, EL emission may not be a beneficial technique to pre-screen devices in order to indicate possible regions of failure.

## 8. GaAs HEMTs

The most commonly reported degradation mechanisms for both GaAs and InP-based HEMTs include contact problems (such as sinking gates in which the gate metal begins to react with the underlying semiconductor), creation of surface states (which can be manifested as what is commonly called gate lag), hot carrier-induced (impact ionization at gate edge), mechanical stress (the absorption of H into Ti-based Schottky gates can lead to compressive stress due to piezo effects in the semiconductor) [3], or avalanche breakdown in the semiconductor, fluorine contamination, and corrosion (mainly related to Al oxidation) [3,4,5,6,7,8,9,10,11,12,13,14,15,16,17,18,19,20,21,22,23,24,25,26,27,28,29,30]. Metamorphic HEMT (MHEMT) technology has been developed using metamorphic buffer layers to grow InAlAs/InGaAs on larger diameter GaAs substrates to overcome the limitations of InP substrates: smaller wafer size, higher cost, and brittle nature. The commercial applications of MHEMTs are predominantly in low noise mm-wave amplifiers for radio communications, automotive collision avoidance radar and high bit-rate fiber systems [18]. The choice of whether they can be used in place of InP-based HEMTs depends upon their DC/RF performance and the chip cost requirements. A number of studies have shown that MHEMTs can exhibit similar reliability to InP HEMTs, with over 10^6^ h mean-time to failure at 125 °C [21,22].

However, the InAlAs/InGaAs MHEMTs require a burn-in step to improve the device stability. Current device designs tend to suffer from device degradation, and a costly burn-in process is typically performed to make the device more stable and eliminate the early degradation when the devices are placed in service. The transistors are generally biased at certain gate and drain voltages for 24–60 h before sending the devices to customers. During the burn-in process, the drain current decreases and Ohmic contact resistance increases with time, and level off around 36 h. Minimizing the burn-in time or eliminating the burn-in step is highly desirable to reduce the device fabrication cost. In order to effectively identify the failure mechanisms, both a high temperature storage test and DC stress were used in this study. Besides MHEMT devices themselves, transmission line method (TLM) patterns were also used to isolate the gate effect on the device degradation.

The MHEMTs were obtained from a commercial vendor. The Ti/Pt-based Schottky gate length was 150 nm, with 1.2 μm spacing between both gate/drain and gate/source. The devices had a two finger design with gate width 75 μm. The TLM patterns also present on the device chip employed 45 μm × 70 μm pads with gaps of 3, 6, 9, 12 and 15 μm.

Typical dc characteristics of the MHEMTs prior to stressing showed the maximum drain-source current density was 0.27 A/mm, with a gate current in the hundreds of nA range. The unity current gain, *f*_t_, was 94 GHz while the maximum frequency of oscillation, *f*_max_, was 124 GHz.

The devices were stressed in one of two ways. Some of the MHEMTs were biased at a source-drain voltage of 3 V for 36 h at 165 °C. Other devices were given a thermal storage test in an oven at 250 °C for 48 h. The dc characteristics were measured before and after both kinds of stressing using an Agilent 4145 parameter analyzer. Some of the devices were also examined by cross-sectional Transmission Electron Microscopy (TEM) to look for reactions of the contacts with the underlying semiconductor. Energy-dispersive X-ray spectroscopy (EDS) elemental analysis was performed to obtain the elemental profiles near the reacted contacts.

To further investigate the origin of the degradation in drain-source current, the sheet resistance and specific contact resistance of the devices were obtained from TLM data as a function of thermal storage time and a function of constant bias voltage stress time. Using the TLMs to examine the increase of the parasitic resistance isolated the effect of the gate sinking on the degradation of drain-source current from the effect of Ohmic metal contact degradation [56,57,58,59,60,61,62]. The total resistance of TLMs increased significantly with time in the first 12 h of thermal storage at 250 °C, while the specific contact resistance increased much more than sheet resistance and showed the contact between Ohmic metal and semiconductor dominated the degradation during the thermal storage. The sheet resistance increased around 18%, while specific contact resistance was reduced by 40%. The device resistance increase was dominated by changes in the sheet resistance instead of contact resistance.

**Figure 11:**
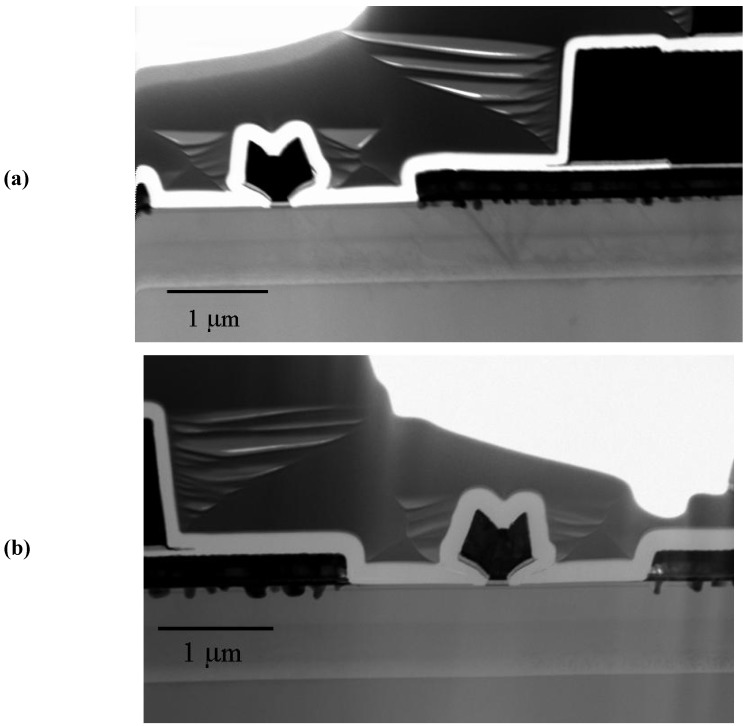
Low magnification cross-section view TEM image of InAlAs/InGaAs MHEMT after storeage at 250 °C for 48 h (**a**) Low magnification cross-section view TEM image of InAlAs/InGaAs MHEMT after DC stress for 36 h (**b**). Reprinted with permission from [62]. Copyright 2010 American Vacuum Society.

TEM cross-sections of a degraded thermal storage HEMT and a constant current stressed HEMT are illustrated in Figure 11a and Figure 11b, respectively. Both samples showed metal spikes with the Ohmic metal diffusing into epitaxial layer, which were formed during the high temperature Ohmic annealing. For the constant current stressed sample, the density of the spikes was higher around the edge of the source Ohmic contact pad and drain Ohmic contact pads, as illustrated in the Figure 11b. Interestingly enough, the region of the high density spikes in the TEM picture matched the estimated transfer length of the TLM measurement as shown in Figure 11b. Thus the formation of the high density spikes could result from the current-induced electromigration.

The drain current density of the commercial power MHEMTS fabricated with the structure described here is around 0.5 A/mm to 1A/mm. In the fabrication, the final metal often has a thickness of 4–6 µm, while the Ohmic metal contact has a thickness of less than 0.3 µm. In these devices, the Ohmic metal is alloyed with the semiconductor and the resistance of the alloyed metal is also larger than the unalloyed metal stack. The 4–6 µm thick final metal does not exhibit a problem with a current density of 0.5 A/mm to 1 A/mm. as shown by TEM, the metal thickness of the region at the edge of the Ohmic contact pad is too thin to sustain the current density to avoid electromigration. During the burn-in process, 20 mA was used to stress a device with gate width of 75 µm (20 mA/75 µm = 266 mA/mm). Therefore, the current density is given as 20 mA/(75 µm × 0.25 µm) = 1 × 10^5^ A/cm^2^, which is the current density through the Ohmic pad. Such a high density of current flowing across the thin Ohmic metal and then across the metal semiconductor interface into the semiconductor causes the Ohmic metal diffusion during the burn-in process. This caused the electromigration-induced voids and the formation of additional metal spikes at the edge of the Ohmic metal contact pads of the source contact pad (left) and drain contact pad (right) after performing the constant current stressing.

**Figure 12:**
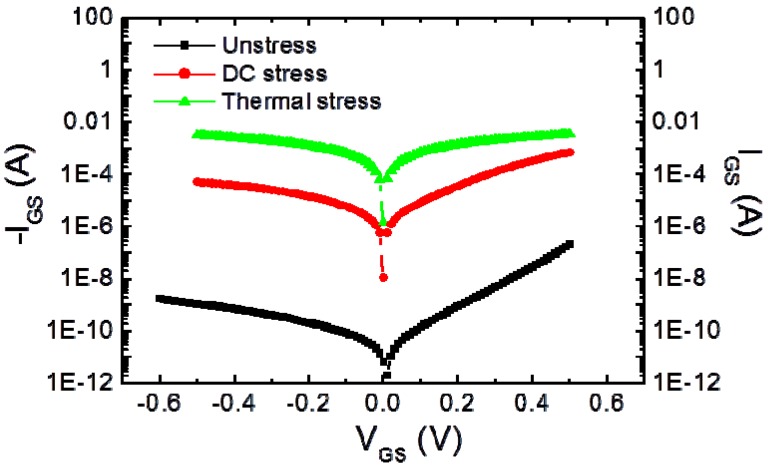
Gate I-V characteristics from MHEMTs before and after thermal or electrical stress.

The gate characteristics of the thermal and DC stressed HEMTs showed significant degradation and gate current increased several orders of magnitude in both forward and reverse bias conditions, as shown in Figure 12. Low magnification cross-section view TEM images (not shown here) of a Pt/Ti/Pt/Au mushroom gate after 165 °C, *V*_DS_ 3V, *J*_DS_ 300 mA/mm for 36 h showed that the bottom Pt of the Pt/Ti/Pt/Au mushroom gate diffused into the InAlAs gate contact layer. EDS elemental analysis was used to analyze the Pt diffusion depth the gate region and showed that around 5–10 nm of Pt diffused into the InAlAs layer.

## 9. GaAs HBTs

The path to commercialization of GaAs-based HBTs saw a number of alterations to the choice of base dopants and emitter layer material [32]. For high current operation, it is necessary to use C as the base dopant, instead of Be or Zn because of its higher doping capability (almost 10^21^ cm^−3^) and superior stability. At high doping levels, Be is not soluble and migration of charged Be interstitials leads to shifts in threshold voltage during device operation. In addition, AlGaAs can be replaced by InGaP, which provides lower surface recombination velocity, reduced susceptibility to oxidation, larger Δ*E*_V_, less recombination in the emitter and improved emitter-base ledge passivation [34,35,36,37,38,39,40,41].

Some reported mechanisms for HBT device degradation under temperature or current density acceleration include midgap trap formation, hydrogen de-passivation from dopants, dislocation propagation, contact degradation, spiking and base dopant diffusion. It was reported that the time to failure could be expressed as [2,33]

(3) TTF=CJ−αe−EαkTj

C is a constant, J is current density,α is current exponent, Ea activation energy, and Tj is the junction temperature. Ea typically exhibits values around ~1 eV [2,33,39]. As we discussed earlier, the initial use of high Be base doping levels led to instability problems. At *p* > 4 × 10^19^ cm^−3^, recombination-enhanced diffusion of Be^+^ interstitials from base into emitter can occur, leading to a positive shift in *V*_BE_ and decrease in gain. Switching to C as the dopant, significantly improved reliability, and extremely high base doping levels could be achieved, with *p* = 3 × 10^21^–10 × 10^21^ cm^−3^. However, even in HBTs with C-doped base layers, there are still issues with metallization stability, surface passivation and avalanche breakdown.

**Figure 13:**
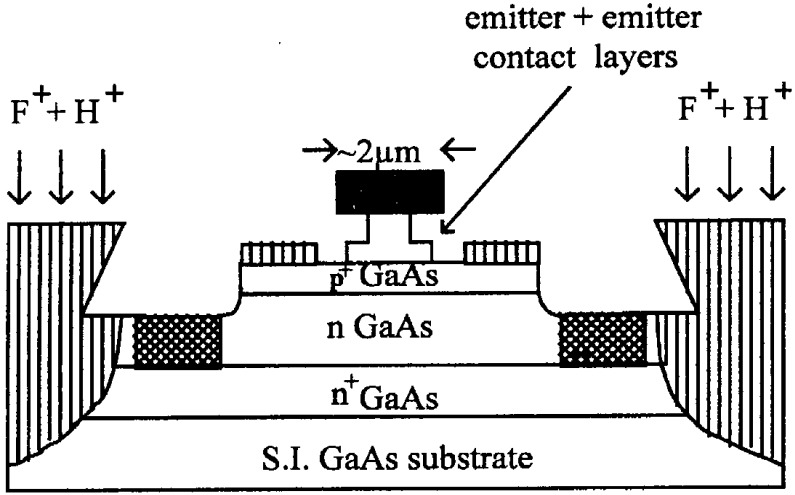
Schematic of implant isolated AlGaAs/GaAs HBT structure.

One important issue arose with the use of implant isolation for maintaining device planarity. A schematic of a typical device is shown in Figure 13. To achieve very high frequency operation, the total layer thicknesses were beyond what could be isolated with conventional energy implantation of oxygen ions and thus a combined implant process that involved oxygen ions and protons to reach the region near the substrate interface, was developed. We have correlated this gain degradation with the presence of hydrogen in the base layer, where it forms electrically inactive C–H complexes. During device operation there is a gradual reactivation of the C acceptors due to minority-carrier enhanced debonding of the hydrogen and this produces a time dependent decrease in current gain. The hydrogen in the base layer may be introduced either during the epitaxial growth from the trimethylgallium or arsine source gases, or during subsequent device fabrication. In the latter case there are two particular opportunities for hydrogen introduction. The first is the implant isolation definition of the active region, normally achieved by a multiple energy F and H implant scheme, followed by annealing to maximize the resistance of the isolation region. We have also found that a second opportunity is the plasma-enhanced chemical vapor deposition of SiN*x* onto the exposed base and emitter mesa sidewall, which is followed by an etch back to leave the nitride sidewall spacer in place.

The typical signature of the effect of hydrogen is a decrease in HBT gain within a few minutes of biasing the device. The forward bias injection of electrons into the base leads to reactivation of carbon acceptors through the reaction

(C–H)^o^ + e^−^ → C^−^ + H^+^ + e^−^ (4)

After this injection-enhanced reactivation of base dopants, the released atomic hydrogen may form molecules (which are electrically inactive), but the effective hole concentration, *p*, increases, so gain decreases. The rate of this process is dependent on injected current density and time.

The rapid initial gain degradation during stress can be linked to a shift of the collector current characteristic to higher *V*_BE_. By comparing the evolution of the collector current shift, Δ*V*_C0_, to that of the base current I_B_ during stress, it is apparent that they share the same dependence in time and on *T*_chuck_ [45]. A gradual decrease in the base sheet resistance during stress was observed, which also occurs on the same time scale as the gain degradation. All the data is consistent with diffusion of hydrogen from the implant isolation region into the base during the anneal to maximize the resistance of this region. The hydrogen passivates some of the carbon acceptors and increases the resulting device gain. As the device is biased and electrons are injected into the base, the carbon acceptors are reactivated and the gain decreases.

An effective solution to this problem is to replace the proton implantation with He^+^ ions. The modified implant isolation schedule still produces a very high resulting sheet resistance of the implanted region as a function of post-implant annealing temperature. Devices fabricated with this scheme did not exhibit the gain degradation [46]. This shows that the main source of the hydrogen was in fact the implant isolation region. When the hydrogen was replaced by He, there was no passivation of the carbon acceptors in the base and therefore no time-dependent gain exhibited by the HBTs.

## 10. Summary

The effect of on-state, high power, step stress was investigated on 0.17 µm gate length HEMTs. Permanent degradation was observed at relatively low drain bias voltages. However, temperature dependent stress tests revealed that permanent degradation was dependent on channel temperatures. Conversely, TLM structures, in which there is no Schottky contact, exhibited exceptional stability up to 25 V bias even though current densities, thus channel temperatures, reached much higher values. Therefore, the Schottky contact is the likely cause for permanent degradation. Breakdown voltages for all three structures indicated that catastrophic failure was not due to channel temperatures, as peak channel temperatures varied significantly (110 °C to 310 °C) at breakdown.

The degradation of AlGaN/GaN HEMTs stressed under high electric field conditions with various device structures and designs were studied. Any residual oxide layer present is seen to be reactive with Ni from the Ni/Au gate metal stack after high reverse gate bias stress. In some instances, oxygen was observed diffusing into the underlying AlGaN layer and further through threading dislocations that terminated below the Schottky contact. Both DC and RF high electric field stress indicated a decrease in Schottky barrier height and large increases in gate leakage current. However, a small decrease in saturation drain current was observed in all instances. Devices with Pt/Ti/Au gate metal scheme showed superior stability up to gate voltages of −100 V. These studies indicate that the reliability of AlGaN/GaN HEMTs can be significantly improved for high electric field applications through the use of more stable gate metals, such as Pt.

In addition, the main degradation mechanisms in AlGaAs/GaAs HEMTs and HBTs have been reviewed. Elevated temperatures, high electric fields and high current densities are all accelerators of degradation. The reaction of the gate metal with the underlying semiconductor, as well as Ohmic contact degradation and creation of surface traps are all issues for HEMTs, while HBTs suffer mainly from contact reaction and junction leakage mechanisms. Careful design of the device geometry is important to avoid high field regions and large current densities through contacts.
